# Genetic architecture of postpartum psychosis: from common to rare genetic variation

**DOI:** 10.1038/s41380-026-03637-w

**Published:** 2026-05-14

**Authors:** Seulgi Jung, Madison Caballero, Adrianna Kępińska, Shelby Smout, Trine Munk-Olsen, Thalia K. Robakis, Veerle Bergink, Behrang Mahjani

**Affiliations:** 1 Seaver Autism Center for Research and Treatment, Icahn School of Medicine at Mount Sinai, New York, NY USA; 2 Department of Psychiatry, Icahn School of Medicine at Mount Sinai, New York, NY USA; 3 Department of Genetics and Genomic Sciences, Icahn School of Medicine at Mount Sinai, New York, NY USA; 4 Research Unit of Child and Adolescent Psychiatry, Department of Clinical Research, University of Southern Denmark, Odense, Denmark; 5 Department of Psychiatry, Erasmus Medical Center, Rotterdam, The Netherlands; 6 Department of Artificial Intelligence and Human Health, Icahn School of Medicine at Mount Sinai, New York, NY USA; 7 Mindich Child Health and Development Institute, Icahn School of Medicine at Mount Sinai, New York, NY USA; 8 Department of Molecular Medicine and Surgery, Karolinska Institutet, Stockholm, Sweden; 9 Department of Medical Epidemiology and Biostatistics, Karolinska Institutet, Stockholm, Sweden

**Keywords:** Neuroscience, Biomarkers

## Abstract

Postpartum psychosis is a severe psychiatric condition marked by the abrupt onset of psychosis, mania, or psychotic depression following childbirth. Despite evidence for a strong genetic basis, the roles of common and rare genetic variation remain poorly understood. Leveraging data from Swedish national registers and genomic data from the All of Us Research Program, we estimated family-based heritability at 55% and whole-genome sequencing-based heritability at 46%. Rare coding variant analysis identified *HMGCR* as a gene in which rare damaging variants confer risk for postpartum psychosis (FDR < 0.05). Analyses of 240,009 participants from the All of Us Research Program and 58,990 participants from the Mount Sinai BioMe Biobank identified significant associations linking deleterious rare variants in *HMGCR* to vascular dementia and mental disorder, not otherwise specified, supporting the gene’s broader psychiatric relevance. Additionally, among the top 200 genes ranked by association statistics, 17% of bipolar disorder, 21% of schizophrenia, and 16–25% of multiple autoimmune disorders exhibit a possible association with postpartum psychosis. These findings reveal unique genetic contributions and shared pathways, providing a foundation for understanding pathophysiology and advancing therapeutic strategies.

## Introduction

Postpartum psychosis is a rare yet profoundly severe psychiatric condition, affecting 0.1–0.2% of mothers following childbirth [1, 2]. It is marked by an acute onset, typically occurring within days to weeks postpartum, and is characterized by a spectrum of symptoms, including mania, depression, psychosis, cognitive disorganization, irritability, and sleep disturbances [3]. These symptoms pose significant risks of suicide and infanticide, necessitating immediate medical intervention. This condition also highlights a striking vulnerability in maternal mental health, with a 10-fold increase in the rate of first-onset psychiatric hospitalizations during this period, encompassing unipolar depressive disorders, schizophrenia, and bipolar affective disorders [4]. Although postpartum psychosis is classified as a bipolar spectrum disorder, its distinct timing, triggers, and associated risks underscore the need for a specialized classification and approach in both research and clinical practice.

The underlying pathophysiology of postpartum psychosis, particularly its genetic basis, remains poorly understood. Emerging evidence points to structural brain changes [5], neuroimmune dysregulation [6, 7], and alterations in the hypothalamic-pituitary-adrenal axis as being associated with this disorder [8]. Genetic contributions to the etiology of postpartum psychosis are becoming increasingly evident. The only genetic study to date used data from women in the Bipolar Disorder Research Network database, comprised of 203 mothers experiencing their first episode of postpartum psychosis and 1225 parous women with a history of bipolar disorder [9]. This study revealed that women with first-onset postpartum psychosis had polygenic risk scores (PRSs) for schizophrenia and bipolar disorder similar to women with bipolar disorder, both higher than controls. Yet, unlike women with bipolar disorder, women with postpartum psychosis did not have elevated PRSs for major depression, suggesting postpartum psychosis may have a distinct genetic etiology. This study highlights the need for more postpartum psychosis-specific studies.

Building on this insight, our recent study leveraging family data from Swedish national registers estimated a relative recurrence risk of 10.69 (95% confidence interval = 6.60 – 16.26) among sisters of mothers diagnosed with their first episode of postpartum psychosis [10]. This rate is higher than the familial recurrence risk for bipolar disorder and schizophrenia, which are 7.9 (95% confidence interval = 7.1 – 8.8) and 9.0 (95% confidence interval = 8.5 – 11.6), respectively, in similar Swedish registry studies [11]. These findings further support the hypothesis that postpartum psychosis has a substantial genetic basis, overlapping with but distinct from other well-studied psychiatric disorders.

The rarity of postpartum psychosis complicates efforts to identify causal variants. Common genetic variants contributing to polygenic risk typically have modest effect sizes, requiring sample sizes, often hundreds of thousands, that are unattainable for rare disorders. Rare deleterious variants have larger per-allele effects, but individual alleles are infrequently observed. Gene-level aggregation methods address this by pooling variants within genes, making rare variant analysis a more practical approach for disorders like postpartum psychosis. A robust method for this is the Transmission and De Novo Association (TADA) model [12], which integrates biological knowledge, pathogenicity metrics, and gene annotations to produce gene-level measures of association evidence that can be transformed into a false discovery rate (FDR).

To elucidate the genetic basis of postpartum psychosis, this study investigates how common and rare genetic variation contribute to its risk. First, we estimate heritability using familial data from Swedish national registers. Next, we estimate heritability from common variants using whole-genome sequencing (WGS) data from the All of Us Research Program [13]. Finally, we apply TADA to test for enrichment of deleterious rare coding variants, identify risk genes for postpartum psychosis, and examine their overlap with genetic risk factors for schizophrenia, bipolar disorder, and multiple autoimmune disorders. Together, these analyses aim to clarify the genetic architecture of postpartum psychosis and provide a foundation for understanding its mechanisms and therapeutic targets.

## Methods

### Study population: Swedish national registers

We utilized data from Swedish national registers to estimate the heritability of postpartum psychosis, following our previous analysis of familial recurrence. Our cohort included women with a first live birth recorded in the Medical Birth Register between January 1, 1980, and October 31, 2017, with follow-up until December 31, 2017. Female full siblings and first-degree cousins were identified through the Multi-Generation Register. Psychiatric diagnoses from inpatient and outpatient care were obtained via the Swedish National Patient Register. Ethical approval and informed consent waiver were granted by the Regional Ethical Review Board in Stockholm.

Postpartum psychosis cases were defined using ICD-8, ICD-9, and ICD-10 codes capturing mania or psychosis within 0-3 months postpartum following the first live birth. This case definition is consistent with prior epidemiological studies demonstrating elevated psychosis risk in the first three months postpartum [1, 4, 10] and with contemporary expert recommendations [14]. Diagnoses included brief psychotic disorder, psychotic disorders not substance- or medically-induced, manic episodes, bipolar disorder, major depressive episodes (severe with psychotic features), recurrent severe depressive episodes with psychotic features, and puerperal psychosis (Table S1; Supplementary Methods). For recurrent depressive disorder codes, cases were included when a depressive episode with psychotic features occurred immediately postpartum, regardless of prior depressive history without psychotic features, consistent with expert consensus that such episodes with clear postpartum onset should be classified as postpartum psychosis [14].

### Calculating heritability from familial concordance

We used a family-based quasi-experimental design to examine genetic and environmental factors, mimicking randomized controlled trials. Using siblings and cousins, we applied Falconer’s Liability Threshold Model to estimate the genetic effect on postpartum psychosis, starting by calculating the familial risk across relationship types (Supplementary Methods).

Briefly, we calculated familial risk, which measures how likely it is that someone with postpartum psychosis also has relatives with the same condition. Then, we compared this familial risk to the condition’s overall prevalence in the general population. This comparison allowed us to estimate how much genetic factors directly contribute to postpartum psychosis. Finally, we combined these estimates across different family relationships, giving more weight to groups with more individuals, to derive overall genetic effects.

### Study population: All of Us

Within the All of Us Research Program (controlled tier, v7) [13], we identified 431 postpartum psychosis cases, of whom 310 had whole-genome sequencing data available. Of these, 268 were classified by ICD-10 code F53.1 (postpartum psychosis), and an additional 163 were identified using criteria from the Swedish registers, encompassing various psychotic disorders occurring within 0–3 months postpartum (Supplementary Methods).

We selected 170,161 female samples without mental disorder diagnoses as potential controls. Of these, 89,333 had whole-genome sequencing data with ancestry principal components available from the All of Us Research Program. Given that direct variant-level quality control and downstream analyses on this full dataset would be computationally intensive, we downsampled to approximately 10,000 ancestry-matched controls. Case-control matching was conducted using the R package ‘optmatch’ based on principal components (PCs) 1–5 provided by the All of Us Research Program, with a 1:30 matching ratio. Euclidean distance metrics were used to select ancestry-matched controls. Using PCs 1–5 captured broad genetic variation due to ancestry differences without excessive adjustment.

To increase the power of the heritability calculation and the rare variant study, we used the currently released WGS data from the All of Us Research Program (controlled tier, v8) [13]. We added 265 independent cases (188 with WGS). From 119,692 eligible female controls, we downsampled to ~5600 ancestry-matched controls using the same PC-based matching procedure. Together, our v7 and v8 All of Us samples contained 461 cases and 4610 controls.

### Analyses of whole genome sequencing data

From the ancestry-matched controls above, we identified 310 cases and 9001 controls with whole-genome sequencing data in the All of Us v7 dataset. We analyzed short-read whole-genome sequencing data from these samples, aligned to GRCh38dh [13]. We excluded samples based on variant count outliers (*n* = 31), low call rates (*n* = 43), close relatedness (*n* = 189), and sex mismatches (*n* = 7). After the sample QC, 305 cases and 8736 controls remained. We further filtered genotypes based on genotype quality, allele balance, read depth thresholds, and Hardy-Weinberg equilibrium (see Supplementary Methods for specific thresholds). These quality control measures ensured high-confidence variant calls for downstream analyses.

Functional annotation used Variant Effect Predictor (VEP) [15]. Variants were classified into protein-truncating variants (PTVs), missense stratified by MPC (Missense Badness, PolyPhen-2, and Constraint) score (MisB, MPC ≥ 2; MisA, 1 ≤ MPC, and synonymous. For PTVs, we applied LOFTEE (Loss-Of-Function Transcript Effect Estimator) filtering, requiring high-confidence designation (see Supplementary Methods). Ultra-rare variants were defined as those with allele count ≤5 in cases/controls from the study cohort and 67,442 samples from gnomAD v3.1 non-psychiatric subset [16]. These ultra-rare variants were used in our subsequent rare-variant analyses.

Following the variant annotation, we excluded 4 cases and 108 controls with rare synonymous variant counts >3 standard deviations above the mean, which could indicate technical issues with their sequencing data. This resulted in our final All of Us v7 analysis cohort of 301 cases and 8628 controls (Supplementary Methods). Using the high-quality data after QC, we conducted 1:10 case-control matching using ‘optmatch’ based on the first five PCs that we calculated using the quality-controlled whole-genome sequencing data, yielding 301 cases and 3010 matched controls (Fig. S1A).

For the version 8 samples, we applied identical sample selection criteria and quality control procedures as those used for the version 7 cohort (detailed in Supplementary Methods). The version 8 samples represent an independent cohort with no overlap with the version 7 participants (160 cases, 1600 controls) (Fig. S1B). Unless otherwise specified, downstream rare-variant burden analyses and TADA were performed using the combined v7 + v8 matched cohort (461 cases, 4610 controls).

### Calculating heritability with whole-genome sequencing data

We estimated heritability using PCGC (Phenotype-Correlation Genotype-Correlation) regression, a method suited for binary traits such as diseases that accounts for ascertainment bias and oversampling [17]. Variants with a minor allele frequency (MAF) >1% from whole genome sequencing data were analyzed. We used both version 7 (301 cases and 3010 controls) and 8 (160 cases and 1600 controls) datasets of the All of Us Research Program, totaling 461 cases and 4610 controls. Because heritability estimates can be biased by population stratification, we restricted the analysis to European ancestry samples, resulting in 198 cases and 2013 controls. We calculated heritability with a prevalence of 0.15% and the first 20 PCs included as covariates (Supplementary Methods).

### Calculating the number of risk genes

Since the All of Us dataset has cases and controls but no parent-offspring trios, we cannot observe true de novo variants. Instead, we approximated them (“de novo-like” events) by treating as “de novo-like” any PTV or damaging missense variant with an allele count of 1 in All of Us and 0 in gnomAD.

We then estimated the total number of postpartum psychosis risk genes using an unseen-species framework [18]. The key insight is that a high proportion of singleton observations suggests many risk genes remain unobserved, while repeated observations of the same genes indicate more complete sampling. We calculated the excess of de novo-like events in cases versus controls to obtain the disease-attributable event count (d), summarized how many genes were hit once versus twice or more among cases (x), and estimated the total number of risk genes (C) using the formula: C = c/u + g^2 * d * (1-u)/u, taking c as the total number of distinct genes with a de novo (d - x), c1 as the count of singleton genes (d - 2x), g as the coefficient of variation in the fractions of genes of each type, and u as 1 - c1/d [19]. Details are provided in the Supplementary Methods.

### Identification of risk genes

We applied the Transmission and De Novo Association (TADA) model to identify risk genes for postpartum psychosis [12], integrating rare coding variants (PTVs, MisB, MisA, Mis0, synonymous) and LOEUF scores [16] using the updated TADA method as developed in large-scale autism exome sequencing studies [20, 21]. Variant counts per gene and LOEUF scores were compiled; however, rare variant counts per gene cannot be shown due to All of Us data policy restrictions.

TADA calculates Bayes factors (BFs) to quantify gene-level associations, incorporating variant counts, sample size, the proportion of risk genes, and variant risk (gamma). Gamma values for PTV, MisB, and MisA variants were determined as the relative risk of variants adjusted by the estimated fraction of genes influencing postpartum psychosis risk. This adjustment was smoothed across LOEUF scores by fitting a logistic regression model within LOEUF bins, yielding rolling-average gamma values. Because MisA variants showed a relative risk lower than 1, gamma MisA was set to 1 (Supplementary Methods).

The total gene-level BF was computed by multiplying BFs across the three variant categories, setting a minimum BF of 1 per category to prevent dilution of evidence from one type by another. These BFs were converted to posterior probabilities, from which p values and multiple-testing-adjusted q values were derived to control the FDR. Genes with an FDR below 0.05 were classified as ‘high-confidence’ risk genes. TADA was applied to the combined v7 and v8 dataset to maximize statistical power.

### Rare variant burden in *HMGCR* and *DNMT1* and related psychiatric disorders

We analyzed the association of rare deleterious variants in *HMGCR* (3-Hydroxy-3-Methylglutaryl-CoA Reductase) or *DNMT1* (DNA Methyltransferase 1) with psychiatric disorders in a cohort of 240,009 WGS samples from the All of Us database [13], excluding postpartum psychosis cases. Psychiatric cases were defined by ICD-10 codes starting with “F” and controls were defined as samples without these codes. Genetic data included rare deleterious variants in the two genes. A burden test using optimal sequence kernel association test (SKAT-O) [22], adjusted for sex and PCs 1-10 to account for ancestry, was conducted with a significance threshold of *p* < 0.00069 (0.05/72), correcting for 72 ICD-10 codes for each gene (Supplementary Methods). For psychiatric disorders with significant p values, we conducted a replication study using whole-exome sequencing data from the Mount Sinai BioMe Biobank, comprising 58,990 samples [23, 24]. For the replication analysis, we applied Bonferroni correction with significance defined as *p* < 0.05 divided by the number of psychiatric disorders with significant p values in the discovery study.

### Comparative analysis

We evaluated the overlap of risk genes for bipolar disorder, schizophrenia, and rheumatoid arthritis with postpartum psychosis using the top 200 and 100 genes from prior exome studies [25–27]. Additionally, we included genes from 29 autoimmune diseases based on availability [27, 28]. The purpose of selecting the top 200 and 100 genes ranked by association statistics is to focus on candidates that have shown some level of association or biological relevance in other studies, even if not all of them reach strict statistical significance thresholds. Using the “propTrueNull” function in the R package limma, we calculated the proportion of true null hypotheses (π0) and estimated the proportion of alternative hypotheses (π1 = 1 - π0), indicating overlapping risk genes (Supplementary Methods). Statistical significance was assessed through 1000 random iterations, comparing observed π1 values with those from random gene sets to calculate an empirical p value. Overlapping risk genes suggest shared pathways between postpartum psychosis and other disorders.

## Results

### Estimating postpartum psychosis heritability through familial concordance

To quantify the genetic contribution to postpartum psychosis, we first estimated heritability using familial concordance data from the Swedish national registers. This approach allowed us to assess overall heritability without relying on direct genetic data. The study cohort included 1,648,759 mothers who experienced their first-ever childbirth between January 1, 1980, and October 31, 2017. Among these, 2514 mothers (0.15%) were diagnosed with postpartum psychosis (Table S2).

Our analysis indicated a heritability of 55% for postpartum psychosis (95% confidence interval, 42–68%). This estimate aligns with heritability estimates for bipolar disorder, which range from 44% to 90%, depending on the study design [11, 29–31].

### Estimating postpartum psychosis heritability through common genetic variation

Building on the family-based heritability estimate, we next quantified the contribution of common (MAF > 1%) genetic variation to postpartum psychosis risk using WGS data. We kept European samples involving 198 mothers with postpartum psychosis and 2013 controls from the All of Us Research Program (Table S3) [13].

Our analysis revealed that autosomal and X-chromosome variants together explain 45.6% ± 22.2% of the phenotypic variation for postpartum psychosis (autosomes: 43.4% ± 22.4%; X chromosome: 2.2% ± 5.5%), providing an estimate of narrow-sense heritability based on inherited common variation. The difference from the estimate derived from Swedish National records suggests that additional heritability may be attributable to rare genetic variants or copy-number variations, which were not captured in this analysis.

### Comparing the burden of rare coding variation between cases and controls

While heritability estimates highlight the overall genetic contribution to postpartum psychosis, they do not identify specific genetic risk factors. To address this, we next investigated the burden of rare coding variants, which may play a critical role in the disorder’s genetic architecture due to their high potential functional impact.

We analyzed rare variants in WGS data from 461 cases and 4610 matched controls sourced from the All of Us Research Program v7 and v8 [13]. We compared the counts of autosomal protein-truncating variants (PTVs; including frameshift, stop-gained, splice donor, splice acceptor, and transcript-ablation variants), missense variants, and synonymous variants (SYNs) in protein-coding genes between cases and controls. Missense variants were further categorized by predicted impact using MPC scores: MisB (MPC ≥ 2, highest probability of deleteriousness), MisA (1 ≤ MPC < 2), and Mis0 (MPC < 1, lowest probability of deleteriousness) [32].

**Fig. 1:**
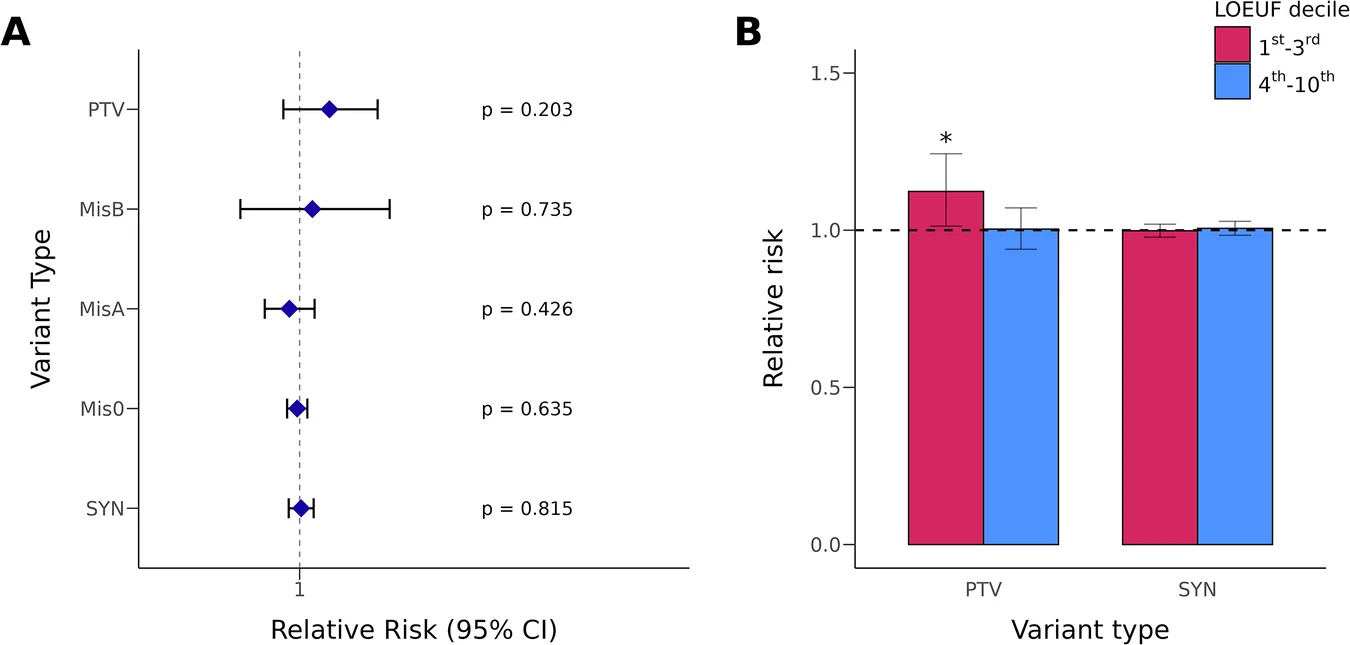
Distribution of rare autosomal protein-coding variants in 461 cases and 4610 controls. **A** Forest plot showing relative risks with 95% confidence intervals (CI) for different variant types. PTV (protein-truncating variant), missense variants, and SYN (synonymous) variants are displayed with their respective p values. Missense variants were categorized using MPC (Missense badness, PolyPhen-2, and Constraint) scores (MisB: variants with MPC ≥ 2, MisA: variants with 2 > MPC ≥ 1, and Mis0: variants with 1 > MPC ≥ 0). The vertical dashed line at 1.0 represents no effect (relative risk = 1). **B** The frequency of PTV and SYN between cases and the unaffected control group in genes stratified by LOEUF (Loss-of-function observed/expected upper bound fraction) score. *p < 0.05 in binomial test.

The average total counts of each variant type were not significantly different between cases and controls in autosomes: PTVs (3.08 in cases vs. 2.97 in controls), MisB (1.19 vs. 1.17), MisA (9.89 vs. 10.02), Mis0 (60.98 vs. 61.16), and SYNs (40.53 vs. 40.57) (Fig. 1A).

Given the lack of differences in overall counts, we examined whether variants were enriched in genes under stronger functional constraints, as is observed in other psychiatric disorders [21, 25, 26, 33, 34]. Using LOEUF score deciles, we grouped genes by constraint [16]. To ensure sufficient statistical power for analysis, we combined deciles 1–3, as deciles 1 and 2 contained too few variants for meaningful testing. We then assessed the burden of PTVs, which are predicted with high confidence to result in loss-of-function. This approach revealed a significant enrichment of PTVs in cases within the most constrained genes (deciles 1–3) (*p* = 0.027, binomial test; Fig. 1B).

To further validate the observed enrichment of PTVs in highly constrained genes (LOEUF deciles 1–3) and to account for potential confounding factors, such as population stratification, we performed a burden test using SKAT-O [22]. We analyzed the burden of rare PTVs in genes within the first to third LOEUF deciles, comparing cases and controls while adjusting for subtle population substructures with the first 50 principal components as covariates. This analysis confirmed that postpartum psychosis cases harbored a significantly elevated burden of PTVs in highly constrained genes (*p* = 0.01).

### Calculating the number of risk genes for postpartum psychosis

The observed enrichment of PTVs in constrained genes among postpartum psychosis cases underscores the potential to identify specific risk genes. As a first step toward systematically identifying these risk genes, we estimated the expected number of protein-coding autosomal genes contributing to postpartum psychosis risk that can be detected using sequencing data.

Since our All of Us dataset lacks the parent-offspring trios needed to identify de novo variants directly, we approximated them by treating as “de novo-like” any PTV or damaging missense variant with an allele count of 1 in All of Us and 0 in gnomAD. We then calculated the excess of these events in cases versus controls and applied an unseen-species estimator to infer the total number of risk genes. Using the combined v7 and v8 dataset (461 cases and 4610 matched controls), this approach yielded a mean estimate of 81 risk genes, representing approximately 0.4% of the 18,128 autosomal protein-coding genes assessed. This estimate of 81 risk genes is substantially lower than corresponding estimates for other psychiatric conditions (e.g., autism, which is estimated to involve 1000 risk genes) [12]. The smaller pool of risk genes implicated in postpartum psychosis mitigates the smaller sample size by increasing the likelihood of observing deleterious variants in the same risk gene among cases.

### Identification of postpartum psychosis risk genes

**Fig. 2:**
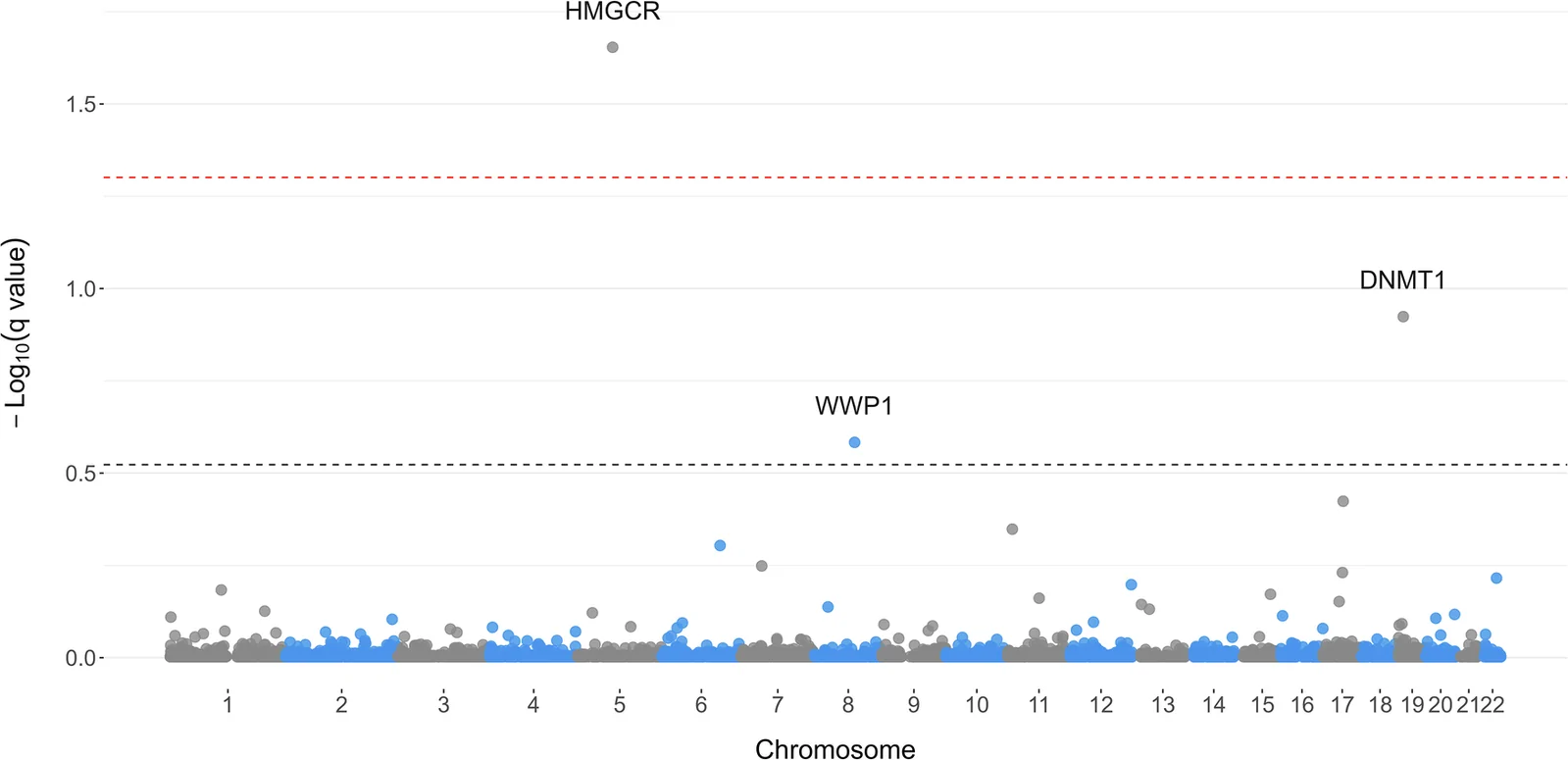
Association of protein-coding genes with postpartum psychosis risk from TADA analysis of the pooled v7 + v8 dataset. Manhattan plot showing -log_10_(q values) for all protein-coding genes across chromosomes. The red dashed line indicates the threshold for q value = 0.05, and the black dashed line represents q value = 0.3. Genes with q value < 0.3 are labeled. Chromosomes are shown in alternating blue and gray colors.

We next sought to identify specific risk genes for postpartum psychosis. We implemented the TADA model, a widely adopted approach for complex disorders that integrates rare loss-of-function variants across multiple variant classes, accounting for evolutionary constraints and the above-estimated number of risk genes [12]. For each autosomal gene, we calculated the strength of association, accounting for different variant types and prior probabilities of relative risk (Table S4). Using the combined v7 and v8 dataset (461 cases and 4610 matched controls), we did not observe inflated significance values, as evidenced by a genomic inflation factor (λ_GC_) of 1.002 and a standard quantile-quantile distribution of p values (Fig. S2). Our analysis identified *HMGCR* as a high-confidence postpartum psychosis risk gene, reaching exome-wide significance (*p* < 2.8 × 10^−6^, Bonferroni-corrected for 18,128 tests) with a q value of 0.02 (Fig. 2 and Table 1). *DNMT1* showed a suggestive signal with a q value of 0.12, not reaching the high-confidence threshold.

**Table 1:** Genes with q value < 0.3 in TADA result.

Gene	pLI	LOEUF	461 cases and 4610 controls					
			OR_PTV_	OR_MisB_	BF_PTV_	BF_MisB_	q value^a^	p^b^
***HMGCR***	**0.999**	**0.260**	**>30**	**20**	**681.04**	**12.87**	**0.022**	**1.23E-06**
*DNMT1*	1.000	0.143	0	25	1.00	721.88	0.119	1.32E-05
*WWP1*	1.000	0.248	>30	>30	8.60	19.33	0.261	4.35E-05

Bold: gene with q value < 0.05.

*BF*, Bayes factor; *LOEUF*, loss-of-function observed/expected upper bound fraction score; *MisB*, missense variant with a MPC (Missense Badness, PolyPhen-2, and Constraint) score ≥ 2; *OR*, odds ratio; *pLI*, the probability of being loss-of-function intolerant score; *PTV*, protein-truncating variant; *TADA*, transmission and de novo association.

^a^q value: false discovery rate, calculated in transmission and de novo association test.

^b^p: *p* value of transmission and de novo association test.

### Association between rare variants in *HMGCR* and *DNMT1* and psychiatric disorders

We sought to determine whether rare deleterious variants (PTV and MisB) in *HMGCR* and *DNMT1* were also associated with a broader spectrum of psychiatric disorders. This analysis aimed to assess the broader relevance of these genes in shared neuropsychiatric pathways and to indirectly validate their biological significance across psychiatric conditions beyond postpartum psychosis. We analyzed 240,009 samples from the All of Us database, excluding postpartum psychosis cases.

Our analysis identified significant associations between *HMGCR* and five psychiatric disorders, with the strongest signals observed for Manic episode (Table S5). For *DNMT1*, we identified significant associations for 12 psychiatric disorders, including emotional disorders with onset specific to childhood. For those psychiatric disorders with significant p values, we performed a replication study using the Mount Sinai BioMe Biobank (*n* = 58,990). Both vascular dementia (ICD-10 code: F01) and mental disorder, not otherwise specified (ICD-10 code: F99) were significantly associated with deleterious rare variants in *HMGCR* with p values < 0.01 (0.05/5) (Table S5). Somatoform disorders (ICD-10 code: F45) showed a significant p value < 0.004 (0.05/12) in the replication study for *DNMT1*. Together, these results suggest that *HMGCR* and *DNMT1* are relevant to psychiatric conditions, which may support their role in postpartum psychosis.

### Comparative analysis of postpartum psychosis and other disorders

Existing studies suggest a close link between postpartum psychosis and both bipolar disorder and schizophrenia [35–43]. Among the 461 mothers with postpartum psychosis in our study cohort, 65.1% had a history of a mental disorder prior to their postpartum psychosis diagnosis (Table S3), which is similar to our recent study, where the rate was 73.0% [10]. Furthermore, growing evidence indicates that immune system changes related to autoimmune disorders during and after pregnancy may contribute to the development of postpartum psychosis [44].

We, therefore, investigated the proportion of risk genes for bipolar disorder, schizophrenia, and 29 autoimmune diseases identified from WGS studies that show evidence of association in the postpartum psychosis TADA result using the “propTrueNull” function in the *limma* package in R [25–27]. Briefly, this approach compares per-gene p values and estimates the proportion of true null hypotheses in the joint, multiple-testing scenario. While individual genes might not achieve significance in isolation, this method identifies their collective impact, particularly when genes share related pathways or biological functions. We performed the analysis using both the top 200 and top 100 genes ranked by association statistics for each disorder.

**Fig. 3:**
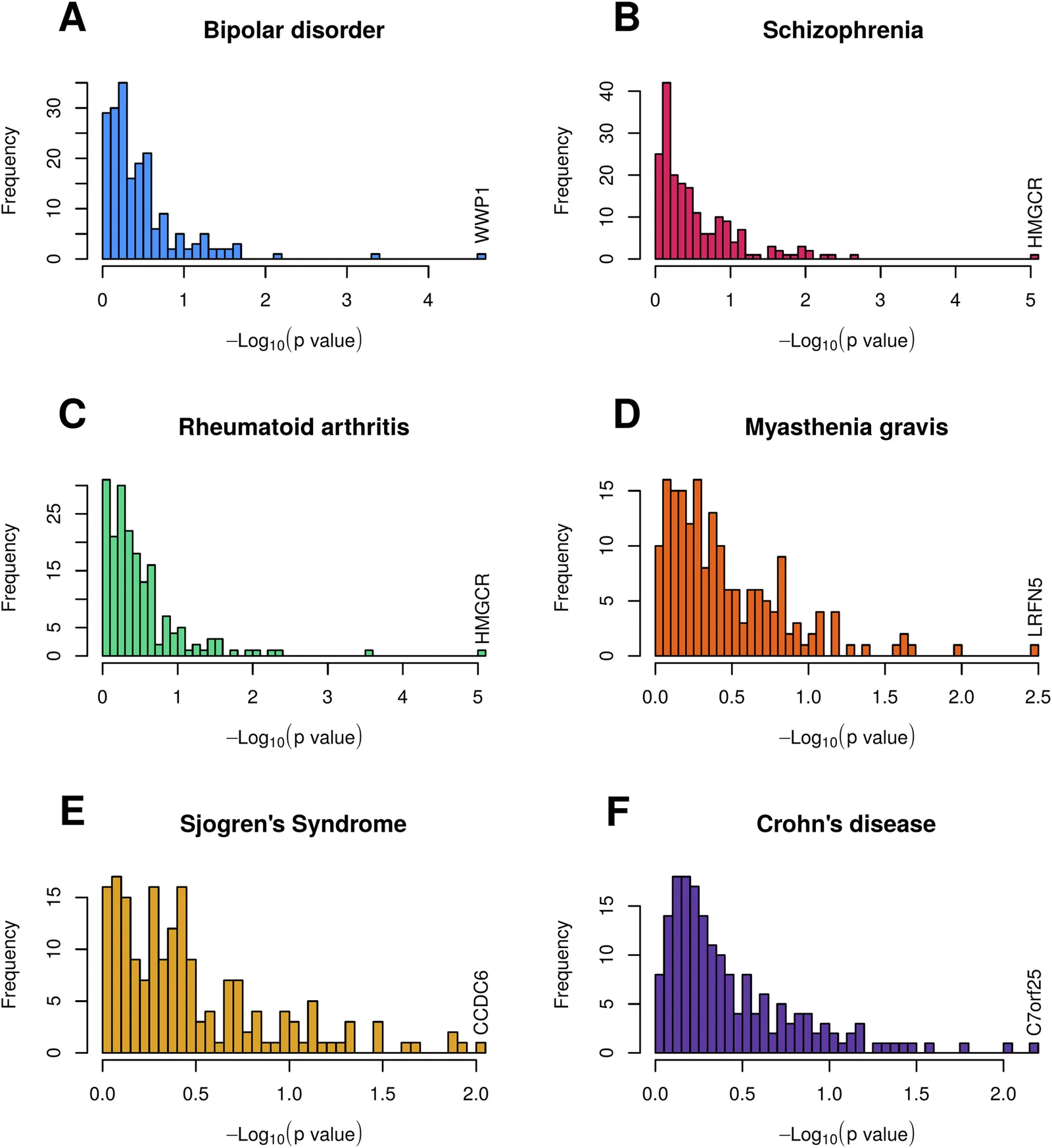
Association of top 200 genes from other psychiatric and autoimmune disorders in postpartum psychosis. The figure shows the TADA association (-log_10_ p values) of the top 200 genes previously reported for each disorder in the postpartum psychosis dataset: **(A)** Bipolar disorder, **(B)** Schizophrenia, **(C)** Rheumatoid arthritis, **(D)** Myasthenia gravis, **(E)** Sjögren’s syndrome, and **(F)** Crohn’s disease. Genes with the lowest p values in postpartum psychosis are labeled. The top 200 genes were ranked by association statistics from each study.

We observed that 17% of the top 200 risk genes ranked by association statistics for bipolar disorder (*P* = 0.019), 21% for schizophrenia (p = 0.023), 16% for rheumatoid arthritis (p = 0.046), 25% for myasthenia gravis (p = 0.010), 20% for Sjögren’s syndrome (p = 0.022), and 18% for Crohn’s disease (p = 0.022) exhibit a possible association with postpartum psychosis (Fig. 3A–F; Table S6). Notably, *HMGCR* (postpartum psychosis p = 9.59 × 10^−6^) emerged as one of the top 200 risk genes ranked by association statistics for both schizophrenia (p = 6.44 × 10^−4^) and rheumatoid arthritis (p = 3.87 × 10^−3^) (Fig. 3B and C). Similarly, *WWP1* (WW domain containing E3 ubiquitin protein ligase 1) (postpartum psychosis p = 2.49 × 10^−5^) was also a bipolar disorder risk gene (p = 6.52 × 10^−3^) (Fig. 3A). When restricting the analysis to the top 100 genes, myasthenia gravis, schizophrenia, and Crohn’s disease remained significant (p < 0.05) and showed higher π₁ estimates, suggesting that the enrichment signal becomes stronger as the analysis focuses on genes with the highest association ranks.

These findings suggest that some risk genes for postpartum psychosis share genetic etiology with other psychiatric disorders. In contrast, others, such as *DNMT1*, were not identified among the top risk genes in recent rare variant studies of bipolar disorder, schizophrenia, and autoimmune diseases.

## Discussion

Postpartum psychosis remains a severely understudied condition despite its substantial risks, affecting 1 in 1000 new mothers and 1 in 5 mothers with bipolar disorder, and occurring at markedly elevated rates among mothers with schizophrenia [45, 46]. This study provides novel insights into its genetic architecture, identifying contributions from both common and rare genetic variants and uncovering overlaps with other psychiatric and autoimmune conditions.

Our family-based analysis estimated heritability at 55% (95% confidence interval, 42%–68%), reflecting primarily autosomal contributions but potentially capturing X-linked inheritance due to the inclusion of female sibling and cousin relationships. This result is comparable with that of bipolar disorder (44–90%) and schizophrenia (64–81%) [11, 29–31, 47, 48].

In comparison, WGS-based analysis estimated heritability at 46% (SE, 22%). The observed differences in heritability estimates likely reflect the challenge of “missing heritability,” as family-based methods capture all inherited genetic effects, including rare, non-coding, and epistatic interactions. At the same time, WGS-based approaches are limited by sample size and coverage of genetic variation.

We next identified a significant enrichment of rare PTVs in highly constrained genes among postpartum psychosis cases, consistent with findings in other psychiatric disorders [20, 21, 25, 26, 34, 49–52]. Using these insights, we estimated that 81 autosomal risk genes contribute to postpartum psychosis, identifiable through sequencing data. This relatively small number compared to other psychiatric conditions, such as autism spectrum disorder (~1000 genes), may enhance the power to detect causal variants in smaller sample sizes.

Among autosomal genes, we identified *HMGCR* as a high-confidence risk gene with an FDR < 0.05. *HMGCR* encodes the rate-limiting enzyme in cholesterol biosynthesis [53, 54]. Low serum cholesterol has been associated with both first-onset psychosis [55] and suicidal behavior [56, 57]. Potential mechanisms include alterations in neuronal membrane viscosity, affecting synaptic transmission, or neuroinflammatory effects resulting from changes in lipid raft composition [57]. In rodent models of Alzheimer’s disease, inhibition of *HMGCR* improved cognitive outcomes and reduced oxidative damage [58]. Moreover, genetic variation in *HMGCR* has been associated with increased risk for neurodegenerative disorders, including Parkinson’s disease [59]. In addition, a recent schizophrenia genome-wide association study identified a variant (rs113537149, *P* = 2.44 × 10^−7^), located 52 kb upstream of *HMGCR* [60], and within the linkage disequilibrium block encompassing *HMGCR*.

The role of *HMGCR* in postpartum psychosis may also relate to pregnancy-induced metabolic changes. Pregnancy significantly elevates serum cholesterol levels, which rapidly return to baseline postpartum [61, 62]. Cholesterol serves as a backbone for steroid hormone synthesis [63], and the extreme hormonal fluctuations associated with childbirth and the postpartum period may interact with disruptions in cholesterol biosynthesis [64, 65], potentially contributing to the pathophysiology of postpartum psychosis.

*DNMT1* showed a suggestive signal (FDR = 0.12). *DNMT1* plays a critical role in maintaining genomic methylation patterns through maintenance methylation [66, 67]. Alterations in its function have pleiotropic effects, with full loss of function generally nonviable [68]. Neuron-specific studies suggest *DNMT1* influences synaptic plasticity and neuronal differentiation [68–70], while human observational studies have linked elevated *DNMT1* expression to bipolar disorder and schizophrenia, though the mechanisms remain unclear [71, 72]. Genetic association studies further implicate *DNMT1* in neuropsychiatric phenotypes; specifically, common variants in *DNMT1* have been correlated with the severity of schizophrenia symptoms [73]. In murine models, postnatal forebrain-specific deletion of *DNMT1* yields anxiolytic and antidepressant-like behavioral phenotypes [74]. Moreover, *DNMT1* mutations in mice disrupt adult neurogenesis and elicit alterations in affective and sensorimotor behaviors [75]. Supporting a broader systemic impact, *DNMT1* mutations are linked to symptoms like hallucinations and cognitive decline, and have been shown to cause mitochondrial stress and abnormal protein handling in cells, similar to what is seen in other neurodegenerative disorders [76, 77]. Beyond neural implications, *DNMT1* deficiency also disrupts immune homeostasis by altering DNA methylation and the function of plasmacytoid dendritic cells [78].

Beyond identifying specific risk genes, our findings reveal genetic overlaps with bipolar disorder, schizophrenia, and autoimmune conditions, emphasizing shared pathways. Notably, *HMGCR* is also one of the top genes for schizophrenia and rheumatoid arthritis, suggesting potential dysregulation in metabolic or immune pathways contributing to the pathogenesis of these conditions. The postpartum period is marked by immune reactivation and hormonal fluctuations, which lead to new onset and relapse of autoimmune diseases. For example, the risk of Myasthenia gravis onset is five times higher in the first six months postpartum compared to during pregnancy [79], and women with Crohn’s disease are more likely to experience disease flare-ups and new-onset psychiatric diagnoses postpartum [80, 81]. Similarly, rheumatoid arthritis has been linked to an increased risk of postpartum psychiatric disorders in women [82], reinforcing the interconnected roles of immune dysregulation and mental health during this critical period.

Despite the rarity of postpartum psychosis, this study validated its findings using multiple complementary approaches. Key strengths include 1) The use of a population-based sample from the Swedish national registers minimizes self-selection bias, ensuring broader representation compared to studies relying on small clinical cohorts, which are often affected by pre-existing psychiatric diagnoses. 2) The combination of family-based heritability estimates with WGS data from the All of Us Research Program provides a complementary approach to understanding the genetic architecture of postpartum psychosis. 3) The study highlights rare coding variants, such as protein-truncating variants, and identifies a specific risk gene, *HMGCR*, offering potential targets for functional research and therapeutic development. 4) The exploration of genetic overlaps with other psychiatric and autoimmune disorders provides broader insights into shared pathways that may contribute to postpartum psychosis and related conditions. Together, these strengths position this study as a significant contribution to the under-researched field of postpartum psychosis genetics.

This study has several limitations: 1) Although postpartum psychosis is one of the narrowest psychiatric phenotypes, its definition varies across studies and databases, potentially affecting comparability. Registry-based data from Swedish national registers and All of Us do not include episode end dates, precluding duration-based sensitivity analyses. 2) We were underpowered to assess genetic risk across different ancestries. 3) The selection of the top 200 and 100 genes from prior studies, including some lacking statistical significance, may dilute signals and reduce the power to detect true overlaps. Predefined gene sets may also introduce false positives, exclude relevant genes, or reflect biases from prior studies with small or unrepresentative samples. 4) We did not stratify participants by psychiatric history, such as distinguishing postpartum psychosis as a continuation of pre-existing disorders from new-onset cases. It cannot be known at the time of onset of a postpartum psychotic episode whether a previously healthy individual will go on to develop bipolar disorder, with additional mood episodes outside the postpartum period, or whether the illness will remain confined to the postpartum period. Incident postpartum psychosis cases may later be diagnosed with bipolar disorder, and in retrospect, the postpartum episode may represent the first onset of bipolar disorder [83, 84]. Onset of schizophrenia postpartum is rare, though misclassification can occur during the acute phase. This limitation may cause our findings to reflect broader patterns of severe mental illness rather than postpartum psychosis-specific factors. Future studies should address this by stratifying participants to improve specificity and clarify overlaps with other conditions. 5) Lastly, rare variant association analysis using TADA lacks direct ancestry adjustment. One solution is to stratify populations by major ancestry groups, estimate TADA parameters separately, and combine Bayes factors, though this approach has limitations for admixed populations with complex genetic structures, as it may not fully capture local ancestry. While we addressed ancestry by matching cases and controls on the first five PCs, this approach minimizes but does not eliminate bias. Future research should incorporate local ancestry inference to address genetic variation in admixed populations better.

In summary, this study provides valuable insights into the genetic architecture of postpartum psychosis, highlighting the contribution to risk captured by common variants through WGS-based heritability estimates, as well as the influence of rare deleterious coding variants identified through gene-based analyses. Further studies using larger samples will be essential to validate these findings and identify additional genetic factors. Functional analyses of identified risk genes, such as *HMGCR* and *DNMT1*, will be critical for elucidating their roles in the disorder and for exploring therapeutic potential. Furthermore, investigating interactions between genetic variants and environmental factors, such as hormonal fluctuations during and after pregnancy, could provide deeper insights into the mechanisms underlying postpartum psychosis.

## Supplementary information

Supplement

## Data Availability

All of Us controlled tier data is available for registered institutions and cohort selection is detailed in the workbench titled “Detecting the prevalence of rare gene mutations.” Data from the Swedish national registers may be obtained from a third party and are not publicly available. Data cannot be shared publicly owing to restrictions by law. Data are available from the National Medical Registries in Sweden after approval by the Swedish Ethical Review Authority.

## Code availability

Code and resources used in this study are available on GitHub at (https://github.com/MahjaniLab/pp_WGS). For any further inquiries or requests for code not available in the repository, please contact the corresponding author.
