# Supplementary material for: Genetic architecture of postpartum psychosis: from common to rare genetic variation
Source: Mol Psychiatry. 2026 May 14;31(10):5528–37. doi: 10.1038/s41380-026-03637-w (PMC13569460; doi:10.1038/s41380-026-03637-w)
Supplement: Supplementary file 1 [file 41380_2026_3637_MOESM1_ESM.docx]

**Supplementary Material**

Genetic Architecture of Postpartum Psychosis:

From Common to Rare Genetic Variation

**Supplementary Methods**

***Study population: Swedish national registers***

We utilized a cohort from the Swedish national registers to estimate the heritability of postpartum psychosis, consistent with our prior analysis of familial recurrence for this disorder. The study included data from the Medical Birth Register with a first-ever live birth between January 1, 1980, and October 31, 2017, with follow-up available until December 31, 2017. Female full siblings and first-degree cousins were identified using the Multi-Generation Register. Psychiatric disorder diagnoses were obtained from the Swedish National Patient Register, which includes both inpatient and outpatient care data. Ethical approval and a waiver of informed consent were granted by the Regional Ethical Review Board in Stockholm, Sweden.

To identify cases of postpartum psychosis, we used ICD-8, ICD-9, and ICD-10 codes to capture all instances of mania and/or psychosis with onset within 0–3 months postpartum following the first live birth. Diagnoses included "brief psychotic disorder," "psychotic disorders not due to a substance or known physiological conditions," "manic episode," "bipolar disorder," "major depressive disorder, single episode, severe with psychotic features," "recurrent depressive disorder, current episode severe with psychotic features," and "puerperal psychosis." A detailed list of the specific ICD codes is provided in Table S1.

***Calculating heritability from familial concordance***

We used a family-based quasi-experimental design that leverages inherent differences in family relationships, such as variations in genetic relatedness and environmental exposure within the same household. This approach allows us to observe the effects of genetic and environmental factors in a quasi-controlled setting, closely mimicking the conditions of randomized controlled trials. Using the pairs of siblings and cousins, we applied Falconer's Liability Threshold Model to quantify the direct additive genetic effect in postpartum psychosis (Table S7). Initially, we computed the familial risk (FR_Postpartum psychosis) — the likelihood that a proband with postpartum psychosis has an affected relative — across the different relationship types. Using formula 25.1.a from Chapter 25 (pages 730-736) of [1], we estimated the regression coefficient (b) between familial risk in relatives of probands with postpartum psychosis and population prevalence for each relationship category:

where P represents the population prevalence of postpartum psychosis, the prevalence of the trait among various types of relationships often mirrors the population prevalence. If this alignment does not occur, we adjust by using the prevalence rate specific to each relationship type to calculate the regression coefficient for that type. Φ and Φ^(-1) denote the standard normal distribution and its inverse, respectively. The regression coefficient (b) for each relationship type is equated to its expected contribution from direct additive genetics to the postpartum psychosis phenotype (Table S8). Subsequently, weighted least squares are employed to estimate the variance components, with weights proportional to the number of probands in each relationship type.

***Study population: All of Us version 7 and 8***

Within the All of Us Research Program (controlled tier, v7), we identified a total of 431 samples with postpartum psychosis. Of these, 268 female samples were classified under the ICD-10 code F53.1 (postpartum psychosis). An additional 163 cases were identified based on the definition used for postpartum psychosis in the Swedish registers. This definition included diagnoses of brief psychotic disorder, psychotic disorders not due to a substance or known physiological condition, manic episodes, bipolar disorder, severe major depressive disorder with psychotic features (single episode or recurrent), and puerperal psychosis, occurring within 0–3 months of childbirth (Table S9). Among the 431 samples, 310 were included in the WGS data.

We defined 170,161 female samples without any SNOMED codes for mental disorders as potential controls. Given the computational burden of performing variant-level quality control and downstream analyses on such a large dataset, we downsampled to 30X (~9,000) ancestry-matched controls to balance computational feasibility with statistical power. Case-control matching was performed using the R package ‘optmatch’, which facilitates optimal matching in observational studies to minimize bias and improve causal inference. Distances between units were calculated using the Euclidean distance metric, and matching was based on principal components (PCs) 1–5, derived from WGS data provided by the All of Us Research Program. From this process, 10,000 candidate ancestry-matched controls were selected. We used 1–5 PCs for the matching as these PCs capture genetic variation due to broad ancestry differences, ensuring effective matching without over-adjusting.

For version 8, we identified 265 additional independent samples with postpartum psychosis, including 173 classified under ICD-10 code F53.1 and 92 identified using the Swedish register definition. Of the 265 samples, 188 had WGS data available. From 119,692 female samples without mental disorder codes, we downsampled to 30X (~5,600) ancestry-matched controls using the same matching procedure.

***Quality control of whole genome sequencing data***

For the version 7 dataset, we analyzed short-read whole genome sequencing data from 310 cases and 9,001 controls from All of Us. Alignment uses the GRCh38dh reference genome. Following All of Us guidelines [1], we excluded 31 individuals with <2.4M or >5.0M total variants, >100k variants not in gnomAD v3.1 [2], or a heterozygous-homozygous variant ratio >3.3. Additionally, we removed 1,039,352 low-quality variants (GQ ≤ 20, DP ≤ 10, and AB ≤ 0.2 for heterozygotes, ExcessHet <54.69, QUAL <60 for SNVs and <69 for short InDels). Our quality control excluded 43 samples with call rates >3 standard deviations below the mean, 189 individuals with close genetic relatedness of kinship value >0.1 in the Kinship-based INference for Genome-wide association studies (KING) [3], and 7 sex-mismatched individuals. After filtering, 305 cases and 8,736 controls were retained. We further filtered out homozygous reference calls with <90% read depth supporting the reference allele or GQ < 25; heterozygous calls with an allele balance <30%, GQ < 25, or >1x10⁻⁹ probability of the allele balance based on a binomial distribution centered on 0.5; and homozygous alternate calls with <90% read depth supporting the alternate allele or GQ < 25. Variants with a call rate ≤90% and Hardy-Weinberg equilibrium p < 1x10⁻¹² were also removed.

We used the Variant Effect Predictor (VEP) to predict the potential functional effect of variants in exons [4]. We classified variants into protein-truncating variants (PTVs: frameshift, stop gained, splice donor, and splice acceptor), missense, and synonymous variants. For PTVs, we applied LOFTEE (Loss-Of-Function Transcript Effect Estimator), requiring an 'HC' designation and no LOFTEE flags except 'SINGLE_EXON.' Missense variants were categorized by MPC scores: MisB (MPC ≥ 2), MisA (2 > MPC ≥ 1), and Mis0 (1 > MPC ≥ 0) [5].

For annotating ultra-rare variants, we included variants with an allele count of ≤ 5 in both the case-control cohort and the non-psychiatric subset of the gnomAD database (v3.1) [2]. We examined the distribution of rare synonymous variants, removing 4 cases and 108 controls with counts >3 standard deviations above the mean. After quality control, there were 301 cases and 8,628 controls.

For version 8, we analyzed short-read whole-genome sequencing from 188 cases and 5,550 controls (All of Us), aligned to GRCh38dh. Using the same All of Us outlier thresholds, we removed 21 individuals. We then applied sample-level QC, excluding low call-rate outliers (>3 SD below the mean; n=89), close relatives (Kinship-based INference for Genome-wide association studies, KING; kinship >0.1; n=130), and sex mismatches (n=6). The final dataset included 160 cases and 5,332 controls. As in version 7, we used identical QC procedures (genotype-level filters, call-rate/HWE filters, and rare-variant definition), unless noted otherwise.

***Calculating heritability with whole genome sequencing data***

We estimated heritability using PCGC (Phenotype-Correlation Genotype-Correlation) regression, an alternative to restricted maximum likelihood (REML) for heritability estimation in binary traits, such as diseases [6]. PCGC estimates heritability on the liability scale, which is more suitable for binary traits, and addresses ascertainment bias by accounting for the bimodal liability distribution and phenotype-covariate correlations introduced by case over-sampling. To calculate heritability using both common and rare variants from whole genome sequencing data, we retained variants with an allele frequency greater than 1% in the matched case-control dataset. We conducted 1:10 case-control matching using 'optmatch' based on the first five PCs that we calculated using the quality controlled whole genome sequencing data, yielding 301 cases and 3,010 matched controls (Fig. S1A). To increase the power of heritability calculation, we performed the same case-control matching using the version 8 data (160 cases and 1,600) (Fig. S1B) and merged them with the version 7 data, totaling 461 cases and 4,610 controls.

To avoid bias from ancestral population differences in PCGC heritability estimates, we restricted the analysis to European ancestry samples (198 cases and 2,013 controls). Genetic ancestry was estimated using a random forest classifier trained on reference populations from the Human Genome Diversity Project (HGDP) and 1000 Genomes Project (N=3,942), using the first 20 principal components derived from high-quality autosomal variants as features. Participants were assigned to ancestry groups based on genetic similarity to reference populations: African (AFR), Americas (AMR), East Asian (EAS), European (EUR), Middle Eastern (MID), and South Asian (SAS). Each sample was assigned to the ancestry with the highest prediction probability (Supplementary Table 3).

We calculated the genetic relationship matrix (GRM) of autosome and X chromosome, separately. Then, we utilized “--mgrm” function of PCGC to add multiple GRMs as input files for calculation of total heritability. We applied a prevalence of 0.15% and included the first 20 PCs as covariates to comprehensively adjust for population structure, particularly when dealing with whole genome sequencing data, where subtle population substructure can influence heritability estimates [7–11]. In this study, we treated the X chromosome as equivalent to autosomes for several reasons. First, our analysis included only female samples, thereby eliminating the differences in copy number between sexes that typically necessitate adjustments for the X chromosome. In females, both X chromosomes are present, and the genetic variance attributable to X-linked loci can be modeled similarly to autosomal loci. Second, by restricting our dataset to a single sex, we bypassed the need to account for dosage compensation mechanisms, as these primarily address differences between males and females.

***Calculating the number of risk genes***

For the rare variant study, we performed 1:10 case-control matching using 'optmatch,' as described above, based on the first five PCs that we calculated using the quality controlled whole genome sequencing data. We did the 1:10 matching because the analysis targets rare deleterious variants with very low event counts, a higher control-to-case ratio increases the precision of background variant rate estimates and stabilizes the calculation of disease-attributable excess events. All controls were selected from the same ancestry-matched pool described above to maintain consistency across analyses.

Since our All of Us dataset consists of case-control data without parent-offspring trios, we could not directly identify de novo variants. We therefore employed an approach to identify de novo-like variants, then used a calculation method to estimate the number of risk genes. We applied stringent filtering criteria to identify de novo-like variants: we retained PTVs or damaging missense variants with allele count = 1 in All of Us and allele count = 0 in gnomAD. We then applied an approach inspired by the "unseen species problem," commonly used in ecological studies [12], and in psychiatric genetics to estimate risk gene counts [13]. This approach infers the expected number of risk-associated genes, including those not yet observed, by analyzing the frequencies of deleterious *de novo* variants and estimating the likelihood that these variants represent a subset of the complete set of risk genes.

We proceeded under the assumption that the number of deleterious variants in cases minus those in controls (d) contributes to the risk of postpartum psychosis, and genes exhibiting recurrent variants (x) were regarded as indicative of risk-associated events.

We applied the formula to calculate the number of risk genes (C): C = c/u + g^2 * d * (1-u)/u, in which: c as the total number of distinct genes with a *de novo* (d - x), c1 as the count of singleton genes (d - 2x), g as the coefficient of variation in the fractions of genes of each type, and u as 1 – c1/d [14]. This calculation was utilized to estimate the number of genes contributing autism spectrum disorder risk [12]. For our calculations, we assume g to be one due to the limited number of observations.

***Identification of risk genes***

We used the Transmission and De Novo Association (TADA) model to identify risk genes for postpartum psychosis [13]. We employed the updated TADA method to integrate rare coding variants (PTVs, MisB, MisA, Mis0, and synonymous variants) and LOEUF scores [2, 15, 16]. Our TADA variant count tallied the number of rare case-control variants for each gene and the LOEUF score for each gene (Table S4). According to the All of Us Data and Statistics Dissemination Policy, we are unable to display the rare variant count for each gene. To provide an overview of the data structure without revealing gene-specific information, we instead present histograms of the number of rare variants per gene for each variant type (PTV, MisB, MisA, and SYN) in Fig. S3A-D. These histograms summarize the distribution of variant counts across all genes included in the TADA model, offering a sense of how many variants contributed to each gene-level analysis.

TADA calculates Bayes factors (BFs) to quantify the statistical strength of gene-level association. This calculation is based on various inputs, including the count of variant events, sample size, proportion of risk genes, and a prior assessment of variant risk (gamma) within each gene. For each gene, gamma for PTVs, gamma for MisB variants, and gamma for MisA variants were calculated as "the relative risk of the variant" divided by "the estimated fraction of genes that substantially influence postpartum psychosis risk," smoothed over the LOEUF score. We created bins of data ordered by LOEUF and fitted a logistic curve to the data. We then computed a rolling-average gamma for PTVs, MisB variants, and MisA variants for the fraction of risk genes, representing the relative enrichment in cases compared to controls (Fig. S4; Table S4). As the relative risk of MisA variants was lower than 1, we set the gamma MisA to 1 (no risk).

To calculate the total gene-level BF, we multiplied the BFs of the three variant classes. To ensure that evidence for association from one variant type is not diluted by evidence against association from another variant type, we set a minimum BF of 1 for each variant class. We then converted BFs to posterior probabilities, which were used to compute p values and q values for adjusting multiple testing, generating a list of candidate risk genes.

We generated gene-level associations using the All of Us whole-genome sequencing controlled tier, version 8 (v8) [17]. Sample selection and quality control mirrored the v7 pipeline. Briefly, we applied identical phenotype definitions for postpartum psychosis, ancestry inference and matching, relatedness filtering, sex checks, contaminated/low-coverage removal, and hard filters on variant and genotype quality. We verified cohort independence by confirming that no v7 participant IDs were present in v8. After 1:10 case-control matching, there were 160 cases and 1,600 controls. To maximize power, we performed a combined analysis by running TADA on a merged dataset constructed from the v7 and v8 cohorts. We defined risk genes by applying widely used statistical thresholds: genes with a FDR below 0.3 were categorized as 'candidate' risk genes, while those with an FDR below 0.05 were considered 'high-confidence' risk genes [15, 16, 18–21].

**Rare variant burden in *HMGCR* and *DNMT1* and related psychiatric disorders**

We investigated which psychiatric disorder is associated with the burden of rare deleterious variants in *HMGCR* or *DNMT1*. The study cohort consisted of 240,009 samples with WGS data from the All of Us research database. Notably, samples with postpartum psychosis were not included in this study cohort. For phenotype classification, cases were defined as samples with an ICD-10 code beginning with "F", which is the mental disorder, while controls were those without the specific ICD-10 code. For instance, if there were 300 samples with the F01 code, the remaining 240,019 samples (240,319 total - 300 cases) served as controls. The genetic data used in this analysis included a genotype matrix of rare deleterious variants, specifically PTV or missense variants predicted to be damaging (MisB), in *HMGCR* or *DNMT1*. We conducted a burden test using SKAT-O, incorporating sex and the first ten PCs (PC1-10) as covariates to account for ancestry [22]. A threshold for statistical significance was set at a p-value of 0.00069 (0.05/72), accounting for the 72 ICD-10 codes beginning with "F" for each gene tested.

For psychiatric disorders showing significant associations in the discovery analysis, we performed replication using whole-exome sequencing data from the Mount Sinai BioMe cohort (n=58,990) [23, 24]. For the variant QC, strict filters were applied: heterozygous genotype calls with allele balance outside 0.30–0.80 were excluded; the retained band showed the expected transition/transversion (Ti/Tv) ratio (~2.7), supporting this filter; variants with a depth of coverage <10X were excluded. Samples were removed for gender discordance between reported sex and genetically inferred sex, insufficient exome breadth of coverage (defined as <80% of targets at 20×), excess contamination (>5%), and unresolved duplicate or near-duplicate submissions. The same phenotype definitions, variant annotations, and statistical approach were applied to ensure consistency across cohorts. For the replication analysis, we applied Bonferroni correction based on the number of psychiatric disorders that showed significant associations in the discovery study (p < 0.05/number of significant discovery findings).

***Comparative analysis***

To assess what proportion of risk genes for bipolar disorder, schizophrenia, and rheumatoid arthritis show evidence of association in postpartum psychosis, we selected the top 200 and 100 genes ranked by association statistics from existing whole-exome sequencing studies [25–27]. We choose 29 autoimmune diseases (alopecia areata, ankylosing spondylitis, asthma, autoimmune hepatitis, autoimmune hypothyroidism, Behcet's disease, celiac disease, Crohn's disease, Graves' disease, Guillain-Barre syndrome, idiopathic thrombocytopenic purpura, lichen planus, multiple sclerosis, myasthenia gravis, myositis, narcolepsy, necrotizing vasculopathies, pernicious anaemia, primary biliary cirrhosis, psoriasis, psoriatic and enteropathic arthropathies, rheumatoid arthritis, sarcoidosis, scleroderma, Sjogren's syndrome, systemic lupus erythematosus, type I diabetes mellitus, ulcerative colitis, vitiligo) based on availability [27, 28]. The purpose of selecting the top 200 and 100 genes is to focus on candidates that have shown some level of association or biological relevance in other studies, even if not all of them reach strict statistical significance thresholds. Genes may not be individually statistically significant but could still contribute meaningfully when considered as a group, especially if they are involved in similar pathways or biological processes. We compared significance using the "propTrueNull" function in the R limma package. The proportion of true null hypotheses (π0) was calculated using a method of "convest" in the propTrueNull function. The proportion of alternative hypotheses (π1) was estimated based on 1 – π0, representing the proportion of risk genes. A π1 >0 indicates that there is a significant portion of hypotheses that are likely true alternatives, suggesting that there are overlapping risk genes between postpartum psychosis and other disorders. To assess the statistical significance of risk gene overlap between postpartum psychosis and the second psychiatric/autoimmune disorder, we performed 1000 iterations of random selection from the total list of autosomal protein-coding genes. In each iteration, we calculated π1 and derived an empirical p value for the observed overlap.

**Fig. S1. Principal component analysis of postpartum psychosis cases and controls across genetic ancestry groups.** **(A)** Version 7 dataset (cases=301, controls=3,010), with each participant assigned to the genetic ancestry group with the highest classifier probability. Cases are represented by triangles and controls by circles. Colors indicate genetic ancestry groups: AFR (African, blue; 77 cases, 773 controls), AMR (Admixed-American, orange; 87 cases, 862 controls), EAS (East Asian, green; 5 cases, 53 controls), EUR (European, red; 129 cases, 1,297 controls), MID (Middle Eastern, grey; 1 cases, 10 controls), and SAS (South Asian, purple; 2 cases, 15 controls). **(B)** Version 8 dataset (cases=160, controls=1,600). AFR (41 cases, 426 controls), AMR (44 cases, 405 controls), EAS (3 cases, 30 controls), EUR (69 cases, 716 controls), SAS (3 cases, 23 controls). Each panel displays pairwise comparisons of the first three principal components: PC1 vs PC2 (left), PC1 vs PC3 (middle), and PC2 vs PC3 (right).

**Fig. S2. Quantile-quantile plots of TADA (Transmission and De Novo Association) analysis p values.** Study cohort: 461 cases and 4,610 controls from All of Us Research Program v7 and v8. The red diagonal line represents the expected distribution of p values if there is no association.

**Fig. S3. Distribution of the number of rare variants per gene used in the TADA model.**

Histograms show the number of variants per gene aggregated across cases and controls for (A) protein-truncating variants (PTVs), (B) missense B variants with MPC ≥ 2 (MisB), (C) missense A variants with 1 ≤ MPC < 2 (MisA), and (D) synonymous variants (SYN). Each panel illustrates the distribution of variant counts across all genes included in the model, providing an overview of the gene-level data structure without revealing gene-specific information, in accordance with All of Us data policy restrictions.

**Fig. S4. Rolling-average prior risk (gamma) of PTV.** This figure shows the case-control gamma PTV (Protein-Truncating Variants) plotted against the LOEUF (Loss-of-function observed/expected upper bound fraction) score. The y-axis represents the gamma value, and the x-axis represents the LOEUF score.

**References:**

1. .	https://support.researchallofus.org/hc/en-us/articles/4617899955092-All-of-Us-Beta-Release-Genomic-Quality-Report. Accessed October 28, 2025.

2.	Karczewski KJ, Francioli LC, Tiao G, Cummings BB, Alföldi J, Wang Q, et al. The mutational constraint spectrum quantified from variation in 141,456 humans. Nature. 2020;581:434–443.

3.	Manichaikul A, Mychaleckyj JC, Rich SS, Daly K, Sale M, Chen W-M. Robust relationship inference in genome-wide association studies. Bioinformatics. 2010;26:2867–2873.

4.	McLaren W, Gil L, Hunt SE, Riat HS, Ritchie GRS, Thormann A, et al. The Ensembl Variant Effect Predictor. Genome Biol. 2016;17:122.

5.	Samocha KE, Kosmicki JA, Karczewski KJ, O’Donnell-Luria AH, Pierce-Hoffman E, MacArthur DG, et al. Regional missense constraint improves variant deleteriousness prediction. bioRxiv. 2017:148353.

6.	Golan D, Lander ES, Rosset S. Measuring missing heritability: inferring the contribution of common variants. Proc Natl Acad Sci U S A. 2014;111:E5272-81.

7.	Malaria Genomic Epidemiology Network. Insights into malaria susceptibility using genome-wide data on 17,000 individuals from Africa, Asia and Oceania. Nat Commun. 2019;10:5732.

8.	Dandine-Roulland C, Bellenguez C, Debette S, Amouyel P, Génin E, Perdry H. Accuracy of heritability estimations in presence of hidden population stratification. Sci Rep. 2016;6:26471.

9.	Liu X, Li YI, Pritchard JK. Trans effects on gene expression can drive omnigenic inheritance. Cell. 2019;177:1022-1034.e6.

10.	Xiong X, James BT, Boix CA, Park YP, Galani K, Victor MB, et al. Epigenomic dissection of Alzheimer’s disease pinpoints causal variants and reveals epigenome erosion. Cell. 2023;186:4422-4437.e21.

11.	Luo C, Liu H, Xie F, Armand EJ, Siletti K, Bakken TE, et al. Single nucleus multi-omics identifies human cortical cell regulatory genome diversity. Cell Genom. 2022;2:100107.

12.	Sanders SJ, Murtha MT, Gupta AR, Murdoch JD, Raubeson MJ, Willsey AJ, et al. De novo mutations revealed by whole-exome sequencing are strongly associated with autism. Nature. 2012;485:237–241.

13.	He X, Sanders SJ, Liu L, De Rubeis S, Lim ET, Sutcliffe JS, et al. Integrated model of de novo and inherited genetic variants yields greater power to identify risk genes. PLoS Genet. 2013;9:e1003671.

14.	Bunge J, Fitzpatrick M. Estimating the Number of Species: A Review. J Am Stat Assoc. 1993;88:364–373.

15.	Satterstrom FK, Kosmicki JA, Wang J, Breen MS, De Rubeis S, An J-Y, et al. Large-Scale Exome Sequencing Study Implicates Both Developmental and Functional Changes in the Neurobiology of Autism. Cell. 2020;180:568-584.e23.

16.	Fu JM, Satterstrom FK, Peng M, Brand H, Collins RL, Dong S, et al. Rare coding variation provides insight into the genetic architecture and phenotypic context of autism. Nat Genet. 2022;54:1320–1331.

17.	All of Us Research Program Investigators, Denny JC, Rutter JL, Goldstein DB, Philippakis A, Smoller JW, et al. The “All of Us” Research Program. N Engl J Med. 2019;381:668–676.

18.	Olfson E, Farhat LC, Liu W, Vitulano LA, Zai G, Lima MO, et al. Rare de novo damaging DNA variants are enriched in attention-deficit/hyperactivity disorder and implicate risk genes. Nat Commun. 2024;15:5870.

19.	Cappi C, Oliphant ME, Péter Z, Zai G, Conceição do Rosário M, Sullivan CAW, et al. De Novo damaging DNA coding mutations are associated with obsessive-compulsive disorder and overlap with Tourette’s disorder and autism. Biol Psychiatry. 2020;87:1035–1044.

20.	Halvorsen M, Samuels J, Wang Y, Greenberg BD, Fyer AJ, McCracken JT, et al. Exome sequencing in obsessive-compulsive disorder reveals a burden of rare damaging coding variants. Nat Neurosci. 2021;24:1071–1076.

21.	Wang S, Mandell JD, Kumar Y, Sun N, Morris MT, Arbelaez J, et al. De Novo Sequence and Copy Number Variants Are Strongly Associated with Tourette Disorder and Implicate Cell Polarity in Pathogenesis. Cell Rep. 2018;24:3441-3454.e12.

22.	Lee S, Emond MJ, Bamshad MJ, Barnes KC, Rieder MJ, Nickerson DA, et al. Optimal unified approach for rare-variant association testing with application to small-sample case-control whole-exome sequencing studies. Am J Hum Genet. 2012;91:224–237.

23.	Abul-Husn NS, Soper ER, Braganza GT, Rodriguez JE, Zeid N, Cullina S, et al. Implementing genomic screening in diverse populations. Genome Med. 2021;13:17.

24.	Belbin GM, Cullina S, Wenric S, Soper ER, Glicksberg BS, Torre D, et al. Toward a fine-scale population health monitoring system. Cell. 2021;184:2068-2083.e11.

25.	Palmer DS, Howrigan DP, Chapman SB, Adolfsson R, Bass N, Blackwood D, et al. Exome sequencing in bipolar disorder identifies AKAP11 as a risk gene shared with schizophrenia. Nat Genet. 2022;54:541–547.

26.	Singh T, Poterba T, Curtis D, Akil H, Al Eissa M, Barchas JD, et al. Rare coding variants in ten genes confer substantial risk for schizophrenia. Nature. 2022;604:509–516.

27.	Zhang J, Fang X, Wu J, Zhang Z, Mu M, Ye D. Identifying therapeutic targets for rheumatoid arthritis by genomics-driven integrative approaches. medRxiv. 2024. https://doi.org/10.1101/2024.03.19.24304536.

28.	Yang L, Ou Y-N, Wu B-S, Liu W-S, Deng Y-T, He X-Y, et al. Large-scale whole-exome sequencing analyses identified protein-coding variants associated with immune-mediated diseases in 350,770 adults. Nat Commun. 2024;15:5924.
